# Imaging synaptic plasticity

**DOI:** 10.1186/1756-6606-4-36

**Published:** 2011-09-29

**Authors:** Zahid Padamsey, Nigel J Emptage

**Affiliations:** 1 Department of Pharmacology, University of Oxford, Mansfield Road, Oxford, OX1 3QT, UK

**Keywords:** synaptic plasticity, long-term potentiation, electrophysiology, optical imaging, FM dyes, Ca^2+ ^imaging, single particle tracking, pHlourins, FRET, FLIM

## Abstract

Over the past decade, the use and development of optical imaging techniques has advanced our understanding of synaptic plasticity by offering the spatial and temporal resolution necessary to examine long-term changes at individual synapses. Here, we review the use of these techniques in recent studies of synaptic plasticity and, in particular, long-term potentiation in the hippocampus.

## Introduction

Traditionally, electrophysiological recordings and biochemical assays have been used to investigate long-term changes in synaptic efficacy following the electrical, pharmacological, or genetic manipulation of synaptic function. These methodologies limit either the spatial or temporal resolution with which biological processes can be observed and manipulated. For example, the electrophysiological characterization of long-term potentiation (LTP), as reported by increases in the amplitude of evoked postsynaptic potentials, often represents a collective change in transmission efficacy across a population of synapses rather than a direct characterization of plasticity at single sites. Moreover, the use of molecular and pharmacological techniques, although useful in elucidating the role of biochemical pathways in the induction and expression of plasticity, reveal little of the spatiotemporal dynamics of cellular signalling that follow synaptic stimulation. Such dynamics, however, are important in understanding how synaptic processing is altered across space and time following the induction of LTP.

In the past decade, advances in optical imaging, combined with the use and development of fluorescent biosensors, have circumvented the problems associated with traditional experimental techniques by offering unparalleled spatiotemporal resolution of molecular events at synapses. Here, we review the use of optical imaging in recent studies of synaptic plasticity in the hippocampus, with a particular focus on how such techniques have been used to assess the 1) presynaptic and 2) postsynaptic expression of LTP, and 3) to examine the spatiotemporal dynamics of plasticity-related signalling.

## Presynaptic expression of LTP

Changes in synaptic efficacy are supported by changes at either pre- or post- synaptic sites [1]. Within the hippocampus, the locus of LTP expression has been a point of contention for many years, in part, due to the difficulties in dissociating the pre- and post- synaptic components of plasticity based on electrophysiological recordings alone [2-8]; many of these difficulties have been overcome by the use of optical imaging.

### FM dyes

**Figure 1:**
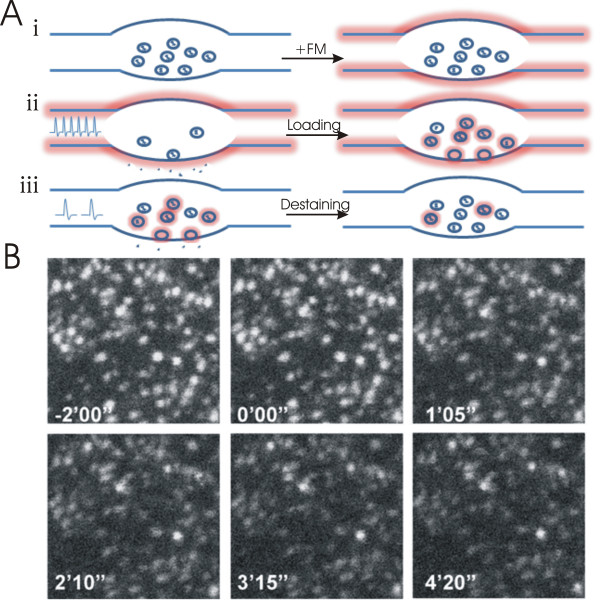
**Imaging vesicle fusion with FM dyes**. (Ai) Bath applied FM dye intercalates with the plasma membrane. (Aii) High frequency stimulation results in vesicular fusion followed by compensatory endocytosis, which generates FM-labelled synaptic vesicles. (Aiii) Following removal of bath applied FM-dye, a subsequent round of lower frequency stimulation results in vesicle fusion and FM-destaining. The loss of fluorescence is used as a measure of presynaptic function at associated boutons. (B) FM dye loaded boutons in the stratum radiatum of an acute hippocampal slice. At time 0 stimulation of the Schaffer-collaterals causes destaining of labelled puncta. Figure 1B: Reprinted from Neuron, 39(6), Zakharenko, S.S., S.L. Patterson, I. Dragatsis, S.O. Zeitlin, S.A. Siegelbaum, E.R. Kandel, and A. Morozov, Presynaptic BDNF required for a presynaptic but not postsynaptic component of LTP at hippocampal CA1-CA3 synapses, p975-90., Copyright (2003), with permission from Elsevier.

The presynaptic component of LTP has been directly investigated by using optical assays of vesicular fusion. One such assay, developed by Betz and Benwick (1992), makes use of fluorescent styryl dyes, such as FM1-43, which readily intercalate with the plasma membrane [9] (Figure 1). During bath application of FM1-43, vesicles generated by endocytosis become dye-labelled. Given that compensatory endocytosis follows activity-dependent vesicle fusion, high-frequency electrical stimulation or elevation of extracellular [K^+^] is used to load synaptic vesicles with dye; FM dye within the extracellular fluid or bound to the plasma membrane is then washed off leaving only intracellular vesicles labelled. During a subsequent round of stimulation (generally at a lower frequency than used for loading), dye is released upon vesicular fusion and detected as a decrease in fluorescence at individual synaptic boutons; the magnitude of this decrease is used as a measure of presynaptic efficacy.

High levels of non-specific binding initially restricted the use of FM-dyes to dissociated hippocampal cultures; in recent years, however, such limitations have been ameliorated with the extracellular application of either cyclodextrin (ADAVSEP) to chelate unbound dye, or sulforhodamine, to quench fluorescence, or the use of multi-photon microscopy to minimise background fluorescence [10-19]. In both dissociated cultures and acute slices from the hippocampus, several groups have demonstrated an NMDAR-dependent enhancement of FM-destaining rate following high-frequency stimulation (≥100 Hz), particularly at boutons with low initial rates of destaining [10,11,15,17,18,20]; conversely, induction of either NMDAR-dependent or mGluR-dependent LTD has resulted in reduced dye-destaining rates [12-14].

By combining FM-imaging with electrophysiological recordings, Zakharenko et al. (2001, 2003) revealed that the induction mechanisms required for the pre- and post-synaptic expression of LTP were partially dissociable [10,11,21]. High-frequency stimulation (200 Hz) of the Schaffer-collaterals readily induced presynaptic changes, - as measured by enhanced FM-destaining rates- the full expression of which was shown to require the activation of L-type voltage-gated calcium channels (L-VGCC) and NMDA-receptors (NMDAR), as well as the release of presynaptic BDNF [10,11,21]. In contrast, low frequency stimulation (50 Hz) failed to produce detectable changes in FM destaining, though, as with 200 Hz stimulation, the amplitude of evoked field potentials were augmented; such changes were dependent on NMDA-receptors and presumably reflected an exclusive enhancement of postsynaptic function [10,11,21]. The pharmacological and electrophysiological dissociation of the pre- and post-synaptic induction of LTP has also been confirmed using pHlourin-tagged synaptophysin (synaptopHlourin) to monitor vesicle fusion (see [22] for details). Although the presynaptic expression of LTP appears to require high-frequency tetanic stimulation, it should be noted that low-frequency pairing (0.33 Hz) of single presynaptic spikes with brief, postsynaptic bursting (3 action potentials at 100 Hz) results in presynaptic potentiation at single synapses [23]; thus high-frequency tetanic stimulation may simply be one way to ensure that postsynaptic bursting is achieved during LTP induction.

One concern with the use of FM-dyes, in addition to their poor signal to noise ratio, is that vesicles and organelles that are not associated with presynaptic terminals can become labelled during dye loading. Without an independent means of confirming the identity of axonal boutons, non-specific labelling has the potential to confound analysis.

### Ca ^2+^sensitive dyes

**Figure 2:**
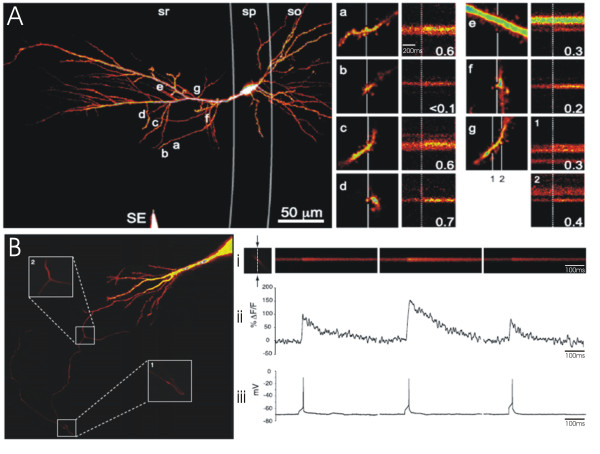
**Optical quantal analysis with Ca^2+ ^sensitive dyes**. (A) Postsynaptic Ca^2+ ^imaging. (Left) Activation of the axons within stratum radiatum (sr) with a stimulating electrode (SE) resulted in stochastic calcium signalling in several spines (a-g) of a dye-filled CA1 neuron in an acute hippocampal slice. (A; a-g) To enable rapid imaging of calcium events over time, laser scanning was restricted to a line (white vertical line) through responsive spines. The results from a line scan depicting successful transmitter release is presented next to each imaged spine; the broken vertical line indicates the point of stimulation. The probability of detecting a calcium event is displayed under each line scan and is used as a measure of the probability of transmitter release at the associated bouton. (B) Presynaptic Ca^2+ ^imaging. (Left) Axonal projection of CA3 hippocampal neuron loaded with a Ca^2+-^sensitive dye. Laser scanning was restricted to a line through an axonal bouton in order to rapidly monitor fluorescence, in response to single action potentials, over time. (i) Three such line scans are presented, along with (ii) the quantified change in fluorescence (ΔF/F) and (iii) the recorded membrane potential. Note that in the second line scan, the action potential generates a larger calcium transient than in the first and second scan. This high-amplitude calcium transient reflects the additional activation of presynaptic NMDAR following the release of glutamate, and can be used as a measure of the probability of transmitter release at the associated bouton. Figure 2A: Reprinted from Neuron, 62(2), Enoki, R., Y.L. Hu, D. Hamilton, and A. Fine, Expression of long-term plasticity at individual synapses in hippocampus is graded, bidirectional, and mainly presynaptic: optical quantal analysis, p242-53., Copyright (2009), with permission from Elsevier. Figure 2B: Reprinted from Neuron, 68(6), McGuinness, L., C. Taylor, R.D. Taylor, C. Yau, T. Langenhan, M.L. Hart, H. Christian, P.W. Tynan, P. Donnelly, and N.J. Emptage, Presynaptic NMDARs in the Hippocampus Facilitate Transmitter Release at Theta Frequency, p1109-27., Copyright (2010), with permission from Elsevier.

In addition to stryl-dyes, Ca^2+ ^indicator dyes have also provided an optical means of assessing transmitter release with single-synapse resolution [23-28]. Single-pulse excitation of afferent fibres, subthreshold for postsynaptic action potential generation, elicit probabilistic Ca^2+ ^transients at stimulated spines (Figure 2A). These transients, although directly generated by Ca^2+ ^influx via NMDARs and voltage-gated channels, are dependent on AMPA-receptor activation and reflect successful release from the presynaptic terminal [24,27]. As such, the likelihood of detecting a Ca^2+ ^transient (P_Ca_) in the spine has been used as a measure of transmitter release probability (P_r_) at the associated bouton [23,25,26,28].

Ca^2+ ^imaging has been used to investigate the expression of LTP at hippocampal synapses [23,25,26,28]. The technique is particularly informative at mossy fibre synapses, where the amplitude of excitatory postsynaptic Ca^2+ ^transients (EPSCaTs) appears to report AMPA receptor activity at individual spines; as such the probability of generating EPSCaTs and the amplitudes of generated EPSCaTs can be used, respectively, to simultaneously monitor the efficacy of both pre- and post-synaptic transmission [26]. Using Ca^2+ ^imaging, Reid et al. (2004) reported that tetanic stimulation (100 Hz) of the mossy fibres increased PCa and led to the emergence of new, high-amplitude Ca^2+ ^events at imaged CA3 synapses; since the distribution of EPSCaT amplitudes remained otherwise unchanged compared to baseline, these high-amplitude events likely reflected the recruitment of additional presynaptic release sites at the synapse rather than an increase in the number of postsynaptic receptors [26]. Consistent with this notion, the authors reported the emergence of evoked Ca^2+ ^events at previously presynaptically silent synapses, which had initially failed to generate EPSCaTs in response to stimulation, even in the absence of extracellular Mg^2+ ^(under Mg2+ free conditions, postsynaptic depolarization is no longer required for NMDAR activation, removing the need for AMPARs in the generation of the EPSCaTs). Accordingly, the authors concluded that LTP was expressed at the presynaptic locus of mossy fibre synapses.

Studies using Ca^2+^imaging at Schaffer-collateral synapses have similarly reported increases in P_Ca _following tetanic stimulation [23,25,28]. However, in contrast to mossy fibre synapses, the amplitude of evoked Ca^2+ ^transients, owing to amplification by Ca^2+^-induced Ca^2+ ^release from internal stores, does not linearly scale with AMPA receptor activation, and so cannot be easily used to examine postsynaptic changes following LTP [24]. However, Enoki et al. (2009) simultaneously and independently assessed both pre- and post- synaptic components of LTP at Schaffer-collateral synapses by combining Ca^2+ ^imaging with electrophysiological recordings [23]. In one set of experiments the authors stimulated a single axon that made synaptic contact with the recorded neuron, and imaged the responsive spine; single-synapse stimulation was confirmed by obtaining a perfect correlation between the successes and failures of EPSCaTs imaged at the spine, and the successes and failures of EPSPs recorded at the soma. Under such conditions, the authors reported that LTP induction increased the probability of evoking an EPSCaT (and its associated EPSP) without altering the magnitude of the EPSP. These observations suggest that the presynaptic terminal is the sole locus for the expression of LTP at Schaffer-collateral synapses. It is, however, important to recognise that Ca ^2+ ^imaging may incur a selection bias since spines associated with large Ca^2+ ^transients are more likely to be detected when the dendrites are searched for synapses responsive to stimulation. It is therefore possible that imaged spines represent a specific subset of synapses that express LTP presynaptically.

In addition to active synapses, Ca^2+^-sensitive dyes have been used to investigate the expression of LTP at silent synapses, traditionally viewed as lacking functional AMPA receptors[28]. AMPARs are required to relieve the Mg^2+ ^block of NMDA receptors, and are thus necessary for the generation of EPSCaTs [24]. Spines, at which Ca ^2+ ^transients can only be evoked in Mg^2+^-free solution, are therefore presynaptically active but postsynaptically silent. Following tetanic stimulation, Ward et al. (2006) demonstrated that EPSCaTs could be generated, in Mg^2+^-containing solution, at previously silent synapses, suggesting the incorporation of functional AMPA recepotrs at these sites [28]. Notably, P_Ca _post-tetanus did not differ from baseline values (calculated in Mg^2+^-free solution), though it increased following a subsequent round of tetanic stimulation. These observations suggest that the locus of LTP expression, whether pre-or post- synaptic, depends on the 'state' of the synapse, whether active or silent.

Although, the detection of Ca^2+ ^events within dendritic spines is generally used to calculate P_r _at Schaffer-collateral synapses, McGuinness et al. (2010) recently demonstrated that a similar measure could be derived by examining the amplitude of evoked Ca^2+ ^events within axonal boutons [29] (Figure 2B). The study reported that single action potentials generate either low or high amplitude Ca^2+ ^events within imaged boutons; whereas low amplitude events were shown to be dependent on Ca^2+ ^influx from voltage-gated channels, high amplitude events additionally required the activation of presynaptic NMDA-receptors by glutamate. Given the dependency of high amplitude events on transmitter release, the authors used the probability of evoking such events as a measure of P_r _at the imaged terminal, and demonstrated that this measure increased following LTP induction. Notably, the use of pre-, as opposed to post-, synaptic Ca^2+ ^events for optical quantal analysis circumvents the observer bias involved in locating EPSCaT-associated spines.

## Postsynaptic expression of LTP

Within the hippocampus, evidence for the postsynaptic expression of LTP has been provided using minimal stimulation and glutamate uncaging paradigms to demonstrate activity-dependent enhancements of responses evoked at single synapses [7,30-35]. Thus, for the past decade there has been a considerable effort to understand the molecular mechanisms underlying the expression of postsynaptic plasticity.

### Single particle tracking

The postsynaptic expression of LTP involves an enhanced sensitivity to glutamate release, for example, by an increase in the number of AMPA receptors at the synapse. New receptors were traditionally thought to originate from an intracellular pool [36], though evidence existed of a second pool of receptors, which were expressed on the plasma membrane but excluded from the postsynaptic density [37]. Recently, the role of these extrasynaptic receptors in synaptic plasticity has been explored using single particle tracking [38,39].

**Figure 3:**
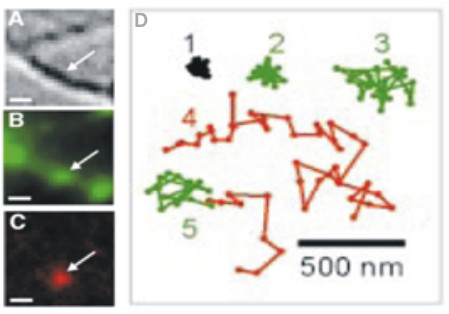
**Tracking lateral movements of single receptors**. (A) Differential interference contrast imaging of a dendrite in neuronal culture. Simultaneous (B) FM1-43 labelling of presynaptic terminals and (C) single particle labelling of the GluR2 subunit of AMPARs by fluorescently-tagged antibodies allows receptor movements to be observed in synaptic and extrasynaptic space. Scale bar = 1 μm. (D) Sample receptor trajectories in synaptic (green) and extrasynaptic (red) space; (D1) Trajectory of fluorescently tagged antibodies fixed on a coverslip. Notice that receptor mobility within a synapse is generally more restricted than movement in extrasynaptic space (D2 & D3 vs.D4). (D5) Trajectory depicting the movement of an extrasynaptic receptor into the synapse. Figure 3: Reprinted by permission from Macmillan Publishers Ltd: [EMBO JOURNAL] (Tardin et al., **22**(18):4656-65) copyright (2011).

Single particle tracking makes use of fluorescently-tagged antibodies to sparsely label proteins in dissociated cell cultures (Figure 3). For surface receptor labelling, antibodies are conjugated to fluorophores such as semiconductor quantum dots, which are quenched within the acidic environment of endocytic vesicles, and are thus only fluorescent when present on the cell surface. Studies also make use of fluorescent markers, such as PSD95 or FM-dye, to delineate synaptic and extrasynaptic space. Tracking of individual AMPA receptors has revealed 1) a mobile pool of synaptic receptors, which constitutively recycle with an extrasynaptic receptor pool, and 2) an immobile pool of synaptic receptors, which are more resistant to exchange [38-41] (Figure 3D).

Notably, receptor trafficking is activity-dependent [40,42-46]. Tardin et al. (2003) reported that the chemical induction of LTP, by bath application of glycine, resulted in a transient increase of synaptic receptor mobility, which was concurrent with a gradual decrease in the number of receptors in the surrounding region, followed by an increase in the proportion of immobile synaptic receptors [41]. Such findings are thought to reflect an activity-driven capture of lateral receptors at the synapse. Consistent with this notion, Heine et al. (2008) demonstrated that high frequency stimulation (50 Hz) greatly reduced the mobility of AMPA receptors, particularly at the synapse, and reduced the number of receptor exchanges between synaptic and extrasynaptic space [45]. The immobilization and accumulation of synaptic AMPA receptors was later shown to be NMDAR-dependent, and to require the activation of CAMKII and, its downstream target, stargazin [42,47]. In contrast to LTP, Tardin et al. (2003) reported that the induction of LTD was associated with an increase in receptor mobility in and around the synapse, perhaps reflecting the diffusion of previously immobilized receptors out of the synapse [41].

Experiments using single particle tracking have proven valuable for understanding activity-dependent receptor movement in synaptic and extrasynaptic space. However, single particle tracking is restricted to neuronal culture and activity-dependent changes in receptor trajectories are examined following global modifications in synaptic efficacy by chemical insults; LTP or LTD examined under these conditions may not be reflective of synaptic plasticity *in vivo*.

### pHlourins

**Figure 4:**
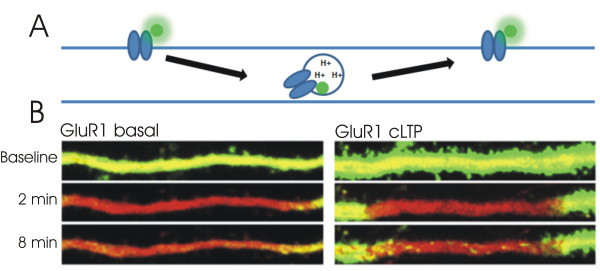
**The use of pHlourin tags to image surface receptors and receptor exocytosis**. (A) pHlourin fluorescence is quenched within the acidic domain of the endosome, ensuring that only surface receptors are imaged. (B) pHlourins report surface GluR1-containing AMPA receptors in a transfected hippocampal neuron in dissociated cell culture. Fluorescence of pHlourin-tagged GluR1 subunits prior to, 2 minutes, and 8 minutes following photobleaching. Fluorescence recovery is mediated primarily by newly exocytosed receptors, as lateral exchange of bleached and unbleached surface receptors is limited under these conditions. Induction of chemical LTP results in a greater number of labelled puncta after photobleaching. Figure 4B: Reprinted from Neuron, 64 (3), Makino, H. and R. Malinow, AMPA receptor incorporation into synapses during LTP: the role of lateral movement and exocytosis, p381-90., Copyright (2009), with permission from Elsevier.

Synaptic transmission, following LTP induction, can be enhanced with receptors supplied either from the extrasynaptic pool, via lateral diffusion, or from the intracellular pool, via exocytosis. To investigate the relative contribution of each receptor pool in the expression of LTP several groups have opted for the use of superecliptic pHlourins (SEP) to label AMPA receptors. SEPs are a pH-sensitive variant of green fluorescent protein (GFP) whose fluorescence is quenched in acidic environments. In contrast to GFP, when SEP if fused to the extracellular domain of AMPA receptor subunits, its fluorescence is only reflective of receptors expressed on the membrane surface, and not of those in acidic, endosomal stores (Figure 4A).

Although SEP fluorescence reports surface AMPA receptors, fluorescence recovery after photobleaching (FRAP) a *large area *of dendrite is reflective of newly exocytosed receptors, since recovery is unlikely to be supported by the lateral diffusion of distant, unbleached receptors. Under such conditions, newly exocytosed receptors are observed as either transient or persistent fluorescent puncta within the imaged area [48] (Figure 4B). Notably, the number of exocytotic events is enhanced following LTP induction [49,50], in a manner dependent on Ras-ERK, but not CAMKII, signalling [50] (Figure 4B). The majority of fusion events occur within the dendrite [48-50], and, given that the machinery for receptor endocytosis/exocytosis appears to be excluded from the postsynaptic density [46,51], even the small proportion of fusion events that occur within the spine are likely to be excluded from the synapse [50]. Regardless of the location of fusion, fluorescence recovery after photobleaching is greater in spines than in the dendrite following LTP induction [49,50], consistent with the notion that newly exocytosed receptors are mobile and can be preferentially "captured" at synaptic sites [46]. However, the extent to which fluorescence recovery after photobleaching is enhanced at spines following LTP induction is modest when compared to the LTP-induced fluorescence increase without photobleaching, which reflects the contribution of both newly exocytosed and pre-existing surface receptors [49,50]. As such, a substantial component of postsynaptic LTP expression appears to be mediated by the synaptic capture of mobile, surface receptors at spines; activity-dependent exocytosis, then, may serve to replenish this mobile pool of surface receptors [46,49].

Fluorescence recovery after photobleaching (FRAP) a *small region *of interest is rapid and, given the low rates of receptor exocytosis, primarily reflects the lateral diffusion of unbleached receptors; the combined use of SEP and FRAP, therefore, can also be used as to measure the average mobility of surface receptors [46,49,50,52,53]. Using this approach, Makino and Malinow (2009) revealed that fluorescence recovery at single spines, 30 minutes after photobleaching, was incomplete in cells expressing the SEP-tagged GluR2, but not GluR1, AMPAR subunit [49]. As such, a portion of GluR2, but not GluR1, containing AMPA receptors appear to be immobilized at the synapse and are resistant to exchange under baseline conditions. Notably, LTP induction, either by glycine application or via glutamate uncaging, resulted in an incomplete recovery of GluR1-SEP fluorescence 30 minutes after photobleaching; fluorescence recovery of GluR2-SEP was unchanged. These findings suggest that GluR1, but not GluR2, containing AMPARs are preferentially captured at the synapse following LTP induction; it is thought that GluR1-containing AMPARs are then later exchanged for receptors containing the GluR2 subunit in an activity-dependent manner [54].

As a general caveat, the use of pHlourins requires overexpression of a given AMPAR subunit (most often GluR1 or GluR2), which may change the number, distribution, and composition of receptors across synaptic, extrasynaptic, and intracellular pools; it remains to be seen whether these changes fundamentally alter the regulation and expression of LTP.

## Synaptic signalling

Although much research has focused on the expression of LTP, with the development and use of new optical reporters, there has been a growing interest in understanding the spatiotemporal dynamics of synaptic signalling that emerge following the induction of LTP, with a particular interest in the compartmentalized nature of synaptic function.

### FRET/FLIM

**Figure 5:**
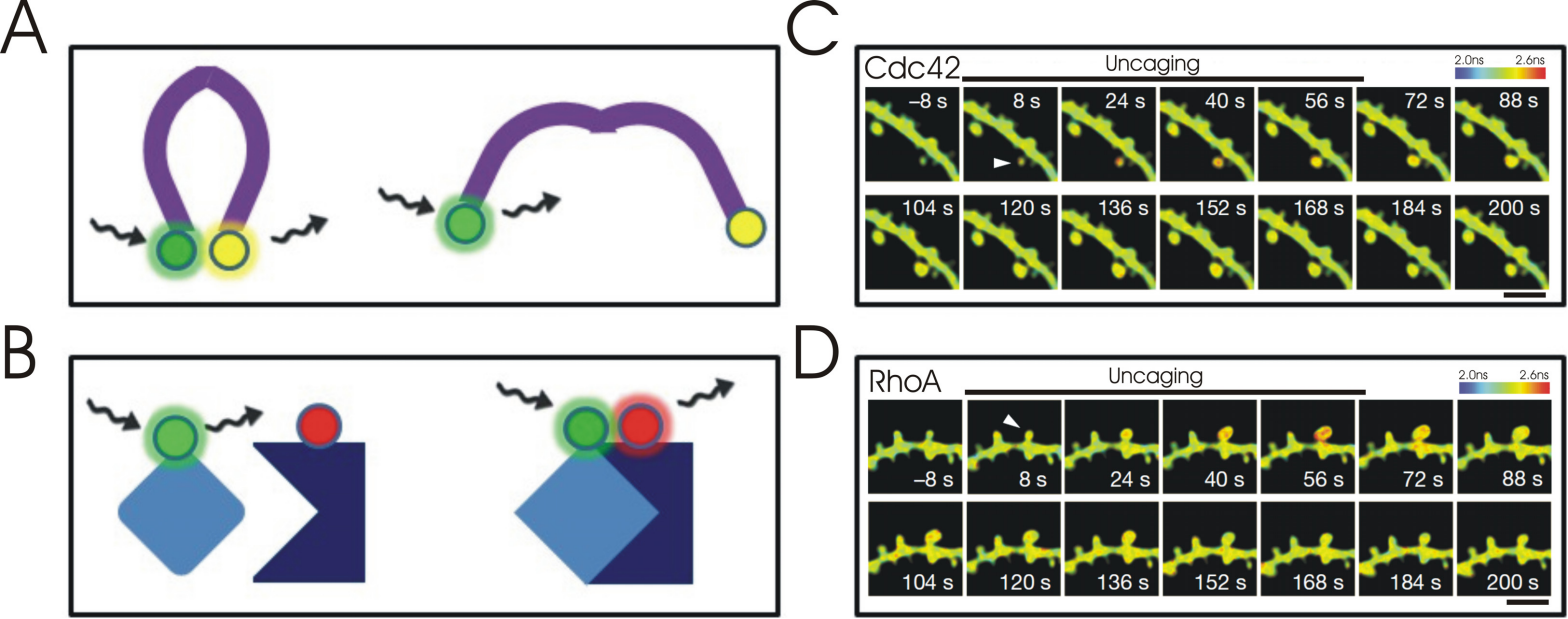
**FRET/FLIM-based reporters of synaptic signaling**. (A) FRET/FLIM-based systems use a donor and acceptor fluorophore to monitor conformational changes within a protein. One such system, which reports CAMKII activity, uses monomeric enhanced GFP (mEGFP) for the donor fluorophore and a variant of YFP for the acceptor fluorophore; the fluorophores are fused, respectively, to the N- and C-terminal tail of the protein. The conformational change that accompanies CAMKII activation increases the distance between the closely apposed N- and C-terminal tails, resulting in a reduction of FRET (YFP/mEGFP fluorescence) and an increase in the fluorescence lifetime of mEGFP; either measure can be used to monitor CAMKII activity. (B) FRET/FLIM based systems can also be designed to report protein-protein interactions. One such system, which reports Ras activation, uses a donor fluorophore (mEGFP) fused to the Ras protein and an acceptor fluorophore (mRFP) fused to a Ras-binding domain; when active, Ras binds to the Ras-binding domain, and the co-localization of the mRFP and mGFP fluorophores results in an increase of FRET (mRFP/mGFPfluorescene), and a decrease in the fluorescence lifetime of mGFP. (C) LTP induction at single spines (arrow head) by repetitive glutamate uncaging (t = 0) is associated with a sustained activation of Cdc42, which remains restricted to the spine and (D) RhoA, which diffuses away from the spine (<10 μm). Figure 5C,D: Reprinted by permission from Macmillan Publishers Ltd: [NATURE] (Murakoshi et al., **472**(7341):100-4) copyright (2011).

In addition to monitoring the distribution of proteins, genetically encoded fluorophores can be engineered to report changes in protein structure or in protein-protein interactions, thus enabling investigators to detect key signalling events in space and time. The detection of such events requires two fluorophores that, when positioned in close spatial proximity (<100Å), participate in fluorescence resonance energy transfer (FRET). The extent to which the spectral profile of photon emission resembles that of the acceptor fluorophore, upon excitation of the donor fluorophore, will depend on the proximity of the two fluorophores, which is sensitive to changes in protein confirmation or protein-protein interaction (see Figure 5 for examples of FRET-based reporters).

Measurements of FRET, however, are difficult to interpret and depend on the concentration of the reporter - a high intensity ratio of acceptor/donor fluorescence, for example, could be generated by a few reporters generating a strong signal or by many reporters generating a weak signal. Such ambiguities can be circumvented by using fluorescence lifetime imaging microscopy (FLIM), which generates images based on the length of time required for fluorescence signals to decay and is, therefore, independent of reporter concentration [55]. When in close proximity to the acceptor fluorophore, the fluorescence of the donor is quenched and the lifetime of its signal is reduced; this reduction can be used to as a measure of FRET.

FRET/FLIM-based reporters have been designed to detect the activation of CAMKII [56,57], ERK [58], Ras [59], RhoA [60], Cdc42 [60], calpain [61], and to examine the organization of the actin cytoskeleton [62]; these reporters have been designed to be sensitive to changes in protein conformation or protein-protein interactions (see Figure 5A, B for examples). FRET/FLIM-based reporters have been instrumental in understanding compartmentalised synaptic signalling following single spine potentiation with glutamate photolysis. Lee et al. (2009), for example, reported that LTP induction resulted in a transient activation of CAMKII (~1 min), which was restricted to the stimulated spine; such compartmentalization was thought to result from the rapid kinetics of CAMKII inactivation, which exceeded the kinetics of CAMKII diffusion out of the spine [57]. Single spine potentiation was also associated with the rapid and long lasting (>30 min) activation of the RhoA and Cdc42, which, respectively, was demonstrated to be necessary for the initiation and maintenance of the spine enlargement that accompanied LTP [60]. Interestingly, despite both proteins having similar diffusion kinetics, Cdc42, but not RhoA, activation remained restricted to the spine (Figure 5C, D). The compartmentalization of Cdc42 signalling, in contrast to that of CAMKII, was suggested to arise from a persistent and spatially restricted activation of Cdc42 within the spine, in combination with rapid deactivation of Cdc42 in the dendrite [60]. RhoA, on the other hand, diffused up to 5 μm along the length of the dendrite; although such diffusive signalling appeared to have no direct effect on neighbouring spine morphology, it may have altered the threshold for activity-dependent structural changes [60]. In fact, such heterosynaptic changes in synaptic metaplasticity have been reported by Harvey et al. (2008). Their study demonstrated that Ras activation, following LTP induction, rapidly diffused from the stimulated synapse into neighbouring spines within a 10 μm radius and remained active for 5-10 min; during this time, the diffuse signalling did not result in a non-specific spread of LTP, but rather, reduced the threshold of stimulation required for LTP induction at neighbouring sites [34,63]. Thus, there exists differing degrees of compartmentalized synaptic signalling that regulate the integration of synaptic activity across space and time.

FRET-based designs allow for the creation of a seemingly limitless number of genetically-encoded optical detectors and will likely be useful for studies *in vivo*. In addition to detecting the activation of various proteins, FRET-based systems have been developed to monitor activity-dependent Ca^2+ ^dynamics, which will also be of tremendous use in future studies of plasticity[64].

### Photoactivation

**Figure 6:**
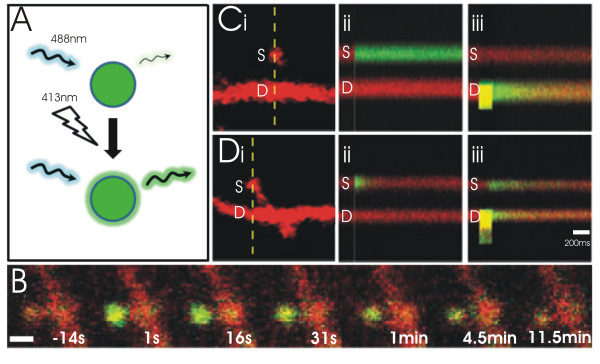
**Photoactivatable GFP enables imaging of a cohort of proteins across space and time**. (A) The fluorescence of photoactivatable green fluorescent protein (paGFP) with 488 nm excitation is stably increased 100-fold after illumination with 413 nm light. (B) Monitoring fluorescence after photoactivation of CAMKII-paGFP in the spine head of a CA1 pyramidal neuron reveals a slow time course of CAMKII diffusion out of the spine. Scale bar = 1 μm. (C, D) Photoactivation of paGFP in the head and underlying dendrite of two spines of a CA1 pyramidal neuron. Laser scanning was restricted to a single line through the spine head and underlying dendrite (broken line in (i)) to enable rapid monitoring of fluorescence over time. paGFP was photoactivated in the spine (ii) or underlying dendrite (iii) at the time denoted the grey vertical line that is present on each line scan. (Ci) An example of a highly compartmentalized spine. (Cii) Fluorescence in the spine was resistant to decay following photoactivation, reflecting the restricted diffusion of activated paGFP out of the spine. (Ciii) Little fluorescence was also observed in the spine after photoactivation in the underlying dendrite, suggesting that the diffusional coupling of the spine and dendrite is limited. (Di) An example of a poorly compartmentalized spine. (Dii) Fluorescence readily decayed in the spine following photoactivation, reflecting the rapid diffusion of activated paGFP out of the spine. (Diii) Fluorescence was also readily detected in the spine after photoactivation in the underlying dendrite, suggesting strong diffusional coupling of the spine and the dendrite (C iii). Figure 6B: Reprinted by permission from Macmillan Publishers Ltd: [NATURE] (Lee et al., **458**(7236):299-304) copyright (2009). Figure 6C,D: From [Bloodgood, B.L. and B.L. Sabatini: Neuronal activity regulates diffusion across the neck of dendritic spines. Science 2005, 310(5749):866-9.]. Reprinted with permission from AAAS.

FRET/FLIM-based reporters elucidate the spatiotemporal profile of protein signalling but cannot be used to independently examine the movements of the protein itself. As such, photoactivation is often used in FRET/FLIM studies to measure the baseline diffusion of a given protein into and out of the spine; such measurements can be important for understanding how protein movement might underlie the spatiotemporal dynamics of its signalling [57,60,63]. Photoactivation uses a photactivatable variant of GFP (paGFP), which is fused to the protein of interest. The fluorescence of paGFP with 488 nm excitation is initially low but stably increases by 100-fold after irradiation with 413 nm light [65] (Figure 6A). In this way, photoactivation can be used to monitor the movements of a spatially and temporally defined cohort of proteins (Figure 6B). paGFP has been fused to many of the synaptic proteins for which FRET-based systems have been designed, including CAMKII [56,57], Ras [63], RhoA and Cdc42 [60]. paGFP has also been used to image the distribution and mobility of PSD95 [66] and actin [67] in single spines during and following LTP induction.

In addition to measuring protein movements, photoactivation has been used to directly examine synaptic compartmentalization. Bloodgood & Sabatini (2005) measured spine/dendrite coupling by activating paGFP in single spines, or their underlying dendrites, and measuring the time constant of equilibration (T_e_) of the resulting fluorescence signal; T_e _varied greatly across spines, ranging from ~100 ms to > 5000 ms [68] (Figure 6C,D). Synaptic compartmentalization was reported to be activity-dependent: it was reduced following chronic incubation with TTX and glutamatergic antagonists, and enhanced following the induction of LTP. Spine neck plasticity has also been explored by measuring the rate of fluorescence recovery after the photobleaching of inert dyes (Alexa) in the spine head [69]. Given that synaptic compartmentalization influences the dynamics of calcium and protein signalling both in the spine and dendrite [57,60,63,67,70,71], the activity-dependent modification of the spine neck may serve as a structural correlate for metaplasticity.

## Conclusion

The examination of cellular processes with single synapse resolution has undoubtedly furthered our understanding of LTP. With the continued improvement and development of optical reporters, and, more generally, in optical imaging techniques, our ever increasing ability to study activity-dependent changes in cellular signalling across space and time will offer new insights into the mechanistic basis of synaptic plasticity.

## Competing interests

The authors declare that they have no competing interests.

## Authors' contributions

ZP drafted the manuscript. ZP and NJE revised the manuscript. ZP and NJE read and approved the final manuscript.
